# Supplementary material for: An all 2D bio-inspired gustatory circuit for mimicking physiology and psychology of feeding behavior
Source: Nat Commun. 2023 Sep 27;14:6021. doi: 10.1038/s41467-023-41046-7 (PMC10533903; doi:10.1038/s41467-023-41046-7)
Supplement: Supplementary file 1 — Supplementary Information [file 41467_2023_41046_MOESM1_ESM.pdf]

Supplementary Information

An All 2D Bio-inspired Gustatory Circuit for Mimicking Physiology and Psychology of Feeding Behavior

Subir Ghosh[1], Andrew Pannone[1], Dipanjan Sen[1], Akshay Wali[2], Harikrishnan Ravichandran[1], and Saptarshi Das[1,2,3,4,]*

[1]Engineering Science and Mechanics, Penn State University, University Park, PA 16802, USA

[2]Electrical Engineering, Penn State University, University Park, PA 16802, USA

[3]Materials Science and Engineering, Penn State University, University Park, PA 16802, USA

[4]Materials Research Institute, Penn State University, University Park, PA 16802, USA

[*] Corresponding author: sud70@psu.edu

Supplementary Information 1

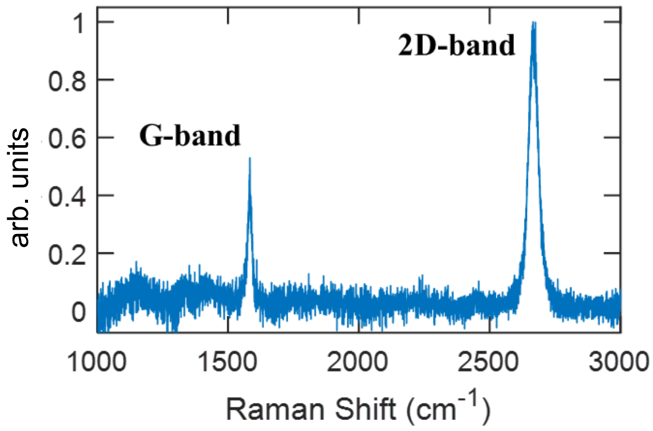

Supplementary Figure 1. Raman spectrum was analyzed for a graphene channel using a laser excitation with a wavelength of 532 nm. The identification of pronounced peaks at approximately 1583 cm^{-1} and 2674 cm^{-1}, which correspond to the G-band and 2D-band, respectively, provides evidence for the existence of a monolayer of graphene.

Supplementary Information 2

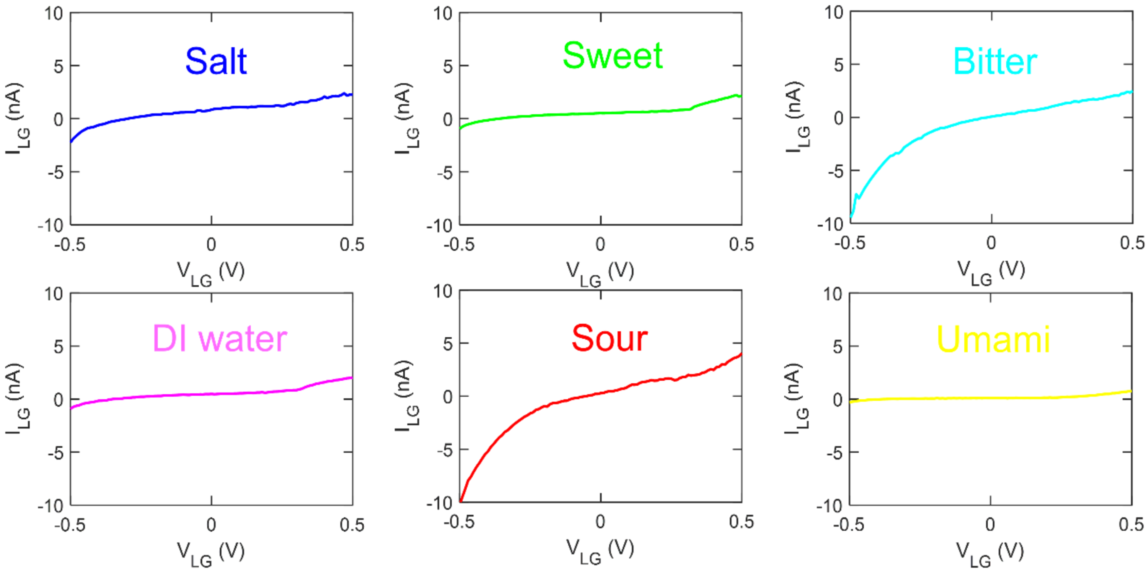

Supplementary Figure 2. Gate leakage current for five different tastes and DI water, as a function of V_{LG}.

Supplementary Information 3

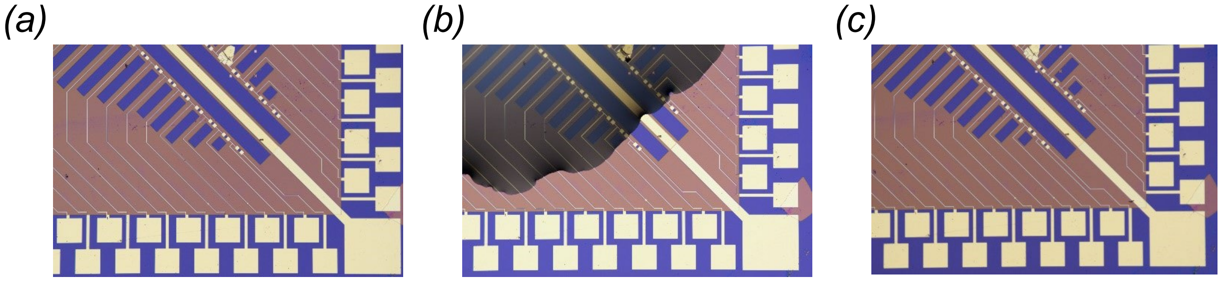

Supplementary Figure 3. Optical images capturing the sample throughout the experiment. **a)** Initial state of the sample before the experiment. **b)** Optical image of the sample showing precipitation of the solution. **c)** Optical image of the sample after thorough wash with DI water, acetone, and IPA.

Supplementary Information 4

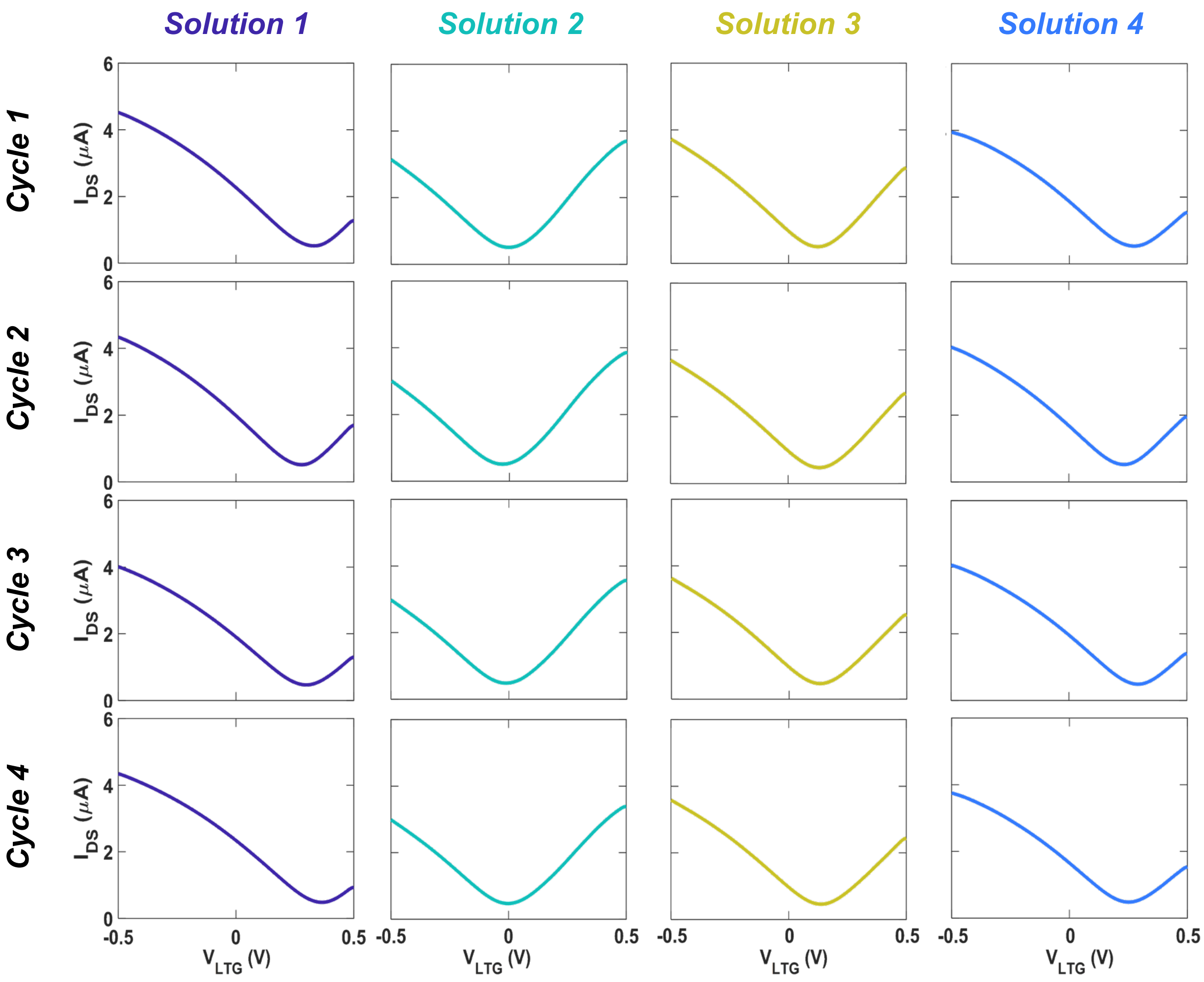

Supplementary Figure 4. The reusability of graphene chemitransistor is shown for four different species for four cycles. The transfer characteristics of four distinct solutions are measured during a single cycle at $V_{DS} = 10$ mV. The transfer characteristics for any species stay mostly unchanged between cycles, according to four rounds of experiments.

Supplementary Information 5

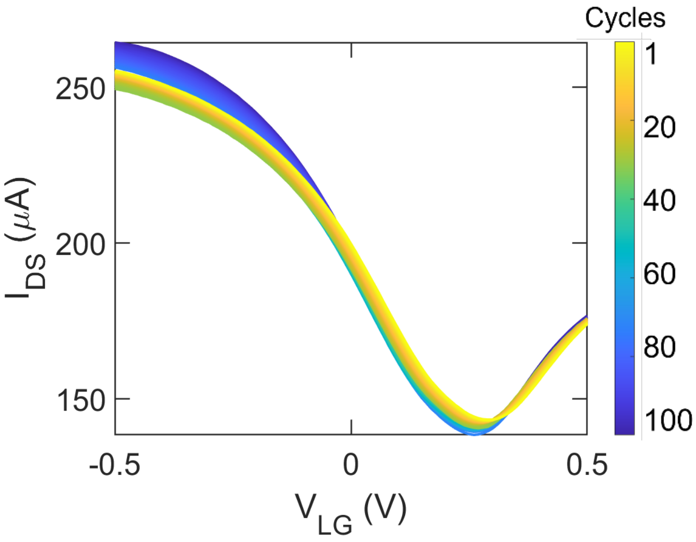

Supplementary Figure 5. Endurance plot of 100 cycles showing consistent transfer characteristics of the graphene chemitransistor for sugar solution at $V_{DS} = 500$ mV.

Supplementary Information 6

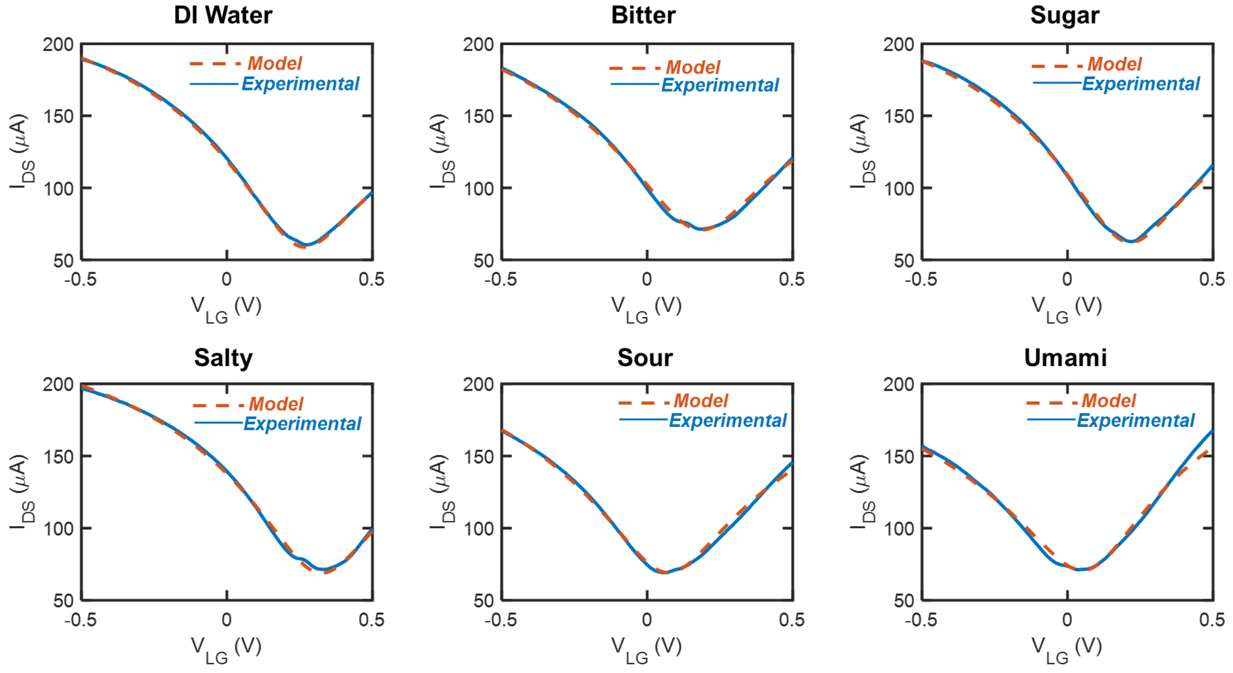

Supplementary Figure 6. To capture the experimental transfer characteristics of graphene chemitransistors we have developed an empirical model where the transition from hole dominant transport to electron dominant transport is captured through a single empirical model as described in Eq. S1

$$I_{DS} = \mu \frac{W}{L} \sqrt{(qn_0)^2 + [C_{OX}(V_{BG} - V_{Dirac})]^2}\, V_{DS} \qquad [S1a]$$

$$\mu = \frac{\mu_n\, exp\left[-\frac{q(V_{BG} - V_{Dirac})}{mk_BT}\right] + \mu_p\, exp\left[\frac{q(V_{BG} - V_{Dirac})}{mk_BT}\right]}{exp\left[-\frac{q(V_{BG} - V_{Dirac})}{mk_BT}\right] + exp\left[\frac{q(V_{BG} - V_{Dirac})}{mk_BT}\right]} = \begin{cases} \mu_n & V_{BG} > V_{Dirac} \\ \mu_p & V_{BG} < V_{Dirac} \end{cases} \qquad [S1b]$$

$$V_{Dirac} = -\, {qn_{imp}}/{C_{OX}} \qquad [S1c]$$

In Eq. S1, q is the charge of an electron, μ is the total carrier field effect (FE) mobility, n_0 is the residual number of carriers in graphene due to the presence of charge puddles in the oxide and energy level broadening due to the coupling with the metal contacts and finite operating temperature, C_{OX} is the oxide capacitance, and V_{Dirac} is the Dirac voltage, which corresponds to the gate voltage where the current is minimum and equally contributed by the holes in the valence band and electrons in the conduction band. Furthermore, k_B is the Boltzmann constant, T is the temperature, and m is a fitting parameter. The expression for μ is defined empirically to capture the asymmetry in the device characteristics originating from the fact that the effective electron mobility (μ_n) and effective hole mobility (μ_p) in our graphene chemitransistors are different.

Supplementary Information 7

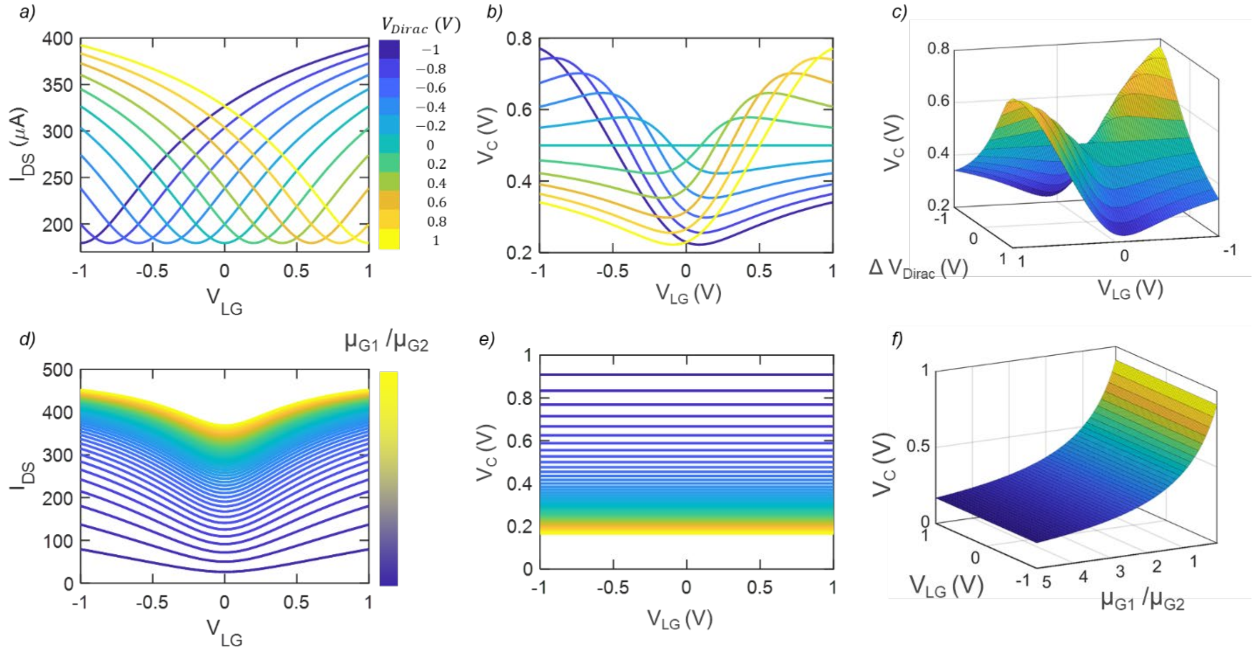

Supplementary Figure 7. Physics-based semi-empirical model for mimicking the graphene based artificial taste receptor. a) Transfer characteristics of a single graphene chemitransistor (GC1) modelled such that its V_{Dirac} falls across a total of 11 different V_{LG} values. b) Output response of two graphene chemitransistors (GC 1 and GC2) in the voltage-divider configuration shown in Fig. 2b. Here, V_{Dirac} of GC2 falls exactly at a V_{LG} of 0V while $\mathrm{V_{Dirac}}$ of $\mathrm{GC_1}$ changes following the same V_{LG} values seen in a). c) A three-dimensional (3D) plot showing V_{C} plotted as a function of V_{LG} and the difference in the V_{Dirac} of GC1 and GC2. d) Transfer characteristics of GC1 modelled for a range of μ_{G1} values. E) Output response of two graphene chemitransistors (GC1 and GC2) in the voltage-divider configuration shown in Fig. 2b. Here, the ratio of the electron and hole mobility, $\left(\frac{\mu_{G1}}{\mu_{G2}}\right)$, changes following the values seen in d) while GC2 remains constant along with its corresponding f) 3D plot showing the relationship of these variables.

Supplementary Information 8

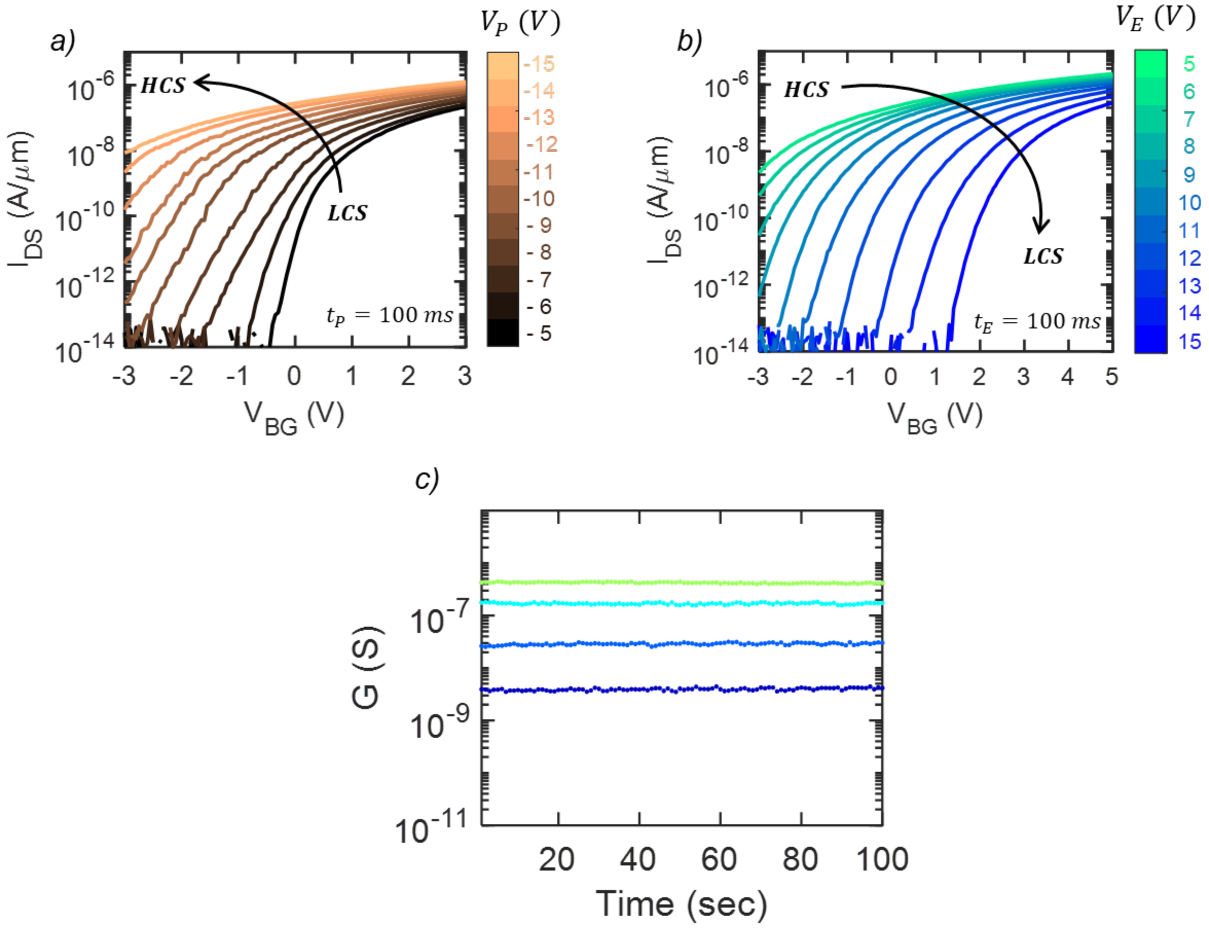

Supplementary Figure 8: Transfer characteristics of a representative MoS_2 memtransistor is shown for different a) programming (V_P) and b) erase (V_P) voltages ranging from 5V to 14V applied to the local-back gate of the device. While application of negative voltage pulses shifts the device threshold voltage (V_{TH}) towards more negative values, positive voltage pulses have the opposite effect where device V_{TH} shifts towards more positive values. c) Retention of four different memory states for a total duration of 100 seconds.

Supplementary Information 9

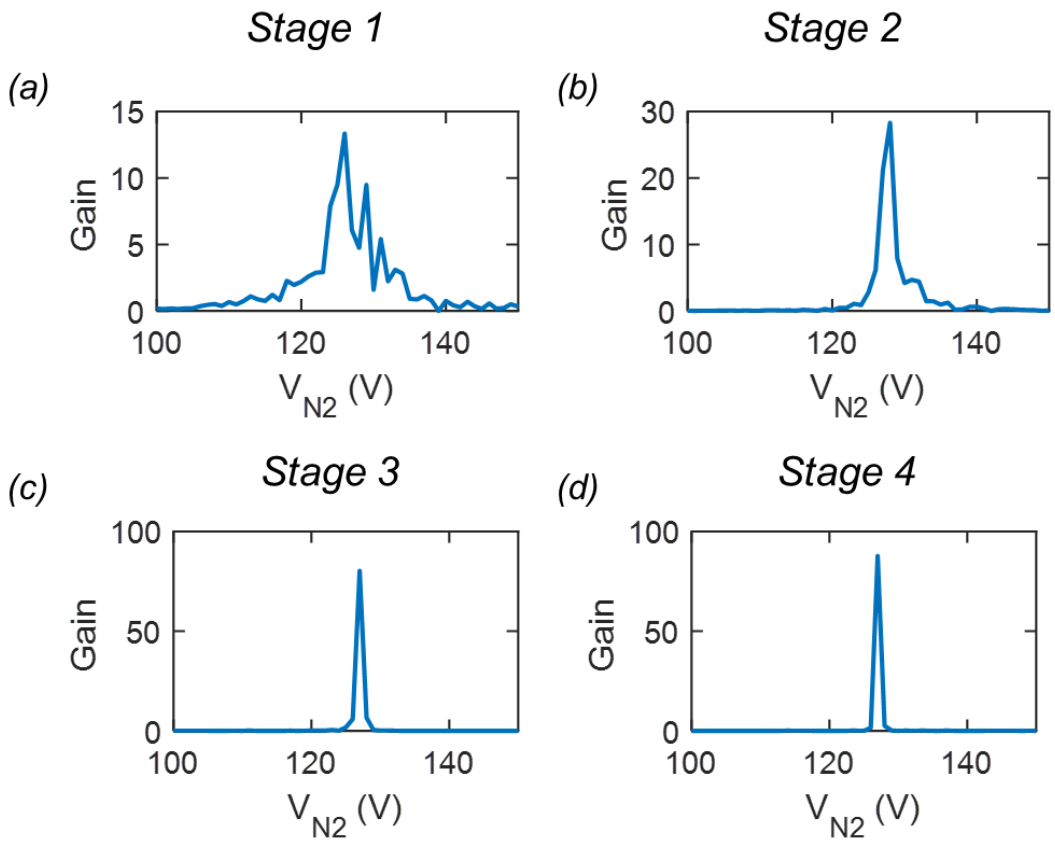

Supplementary Figure 9. Detailed view of the gain plots for all four stages of the inverter, allowing for a comprehensive understanding of the inverter's amplification behavior. The peak gain values of the four stages of the inverter increase steadily in a monotonous fashion. The first stage of the circuit provides a peak gain of approximately 14. Moving on to (b) the second stage, the peak gain value is about 29, (c) the third stage provides a higher gain of approximately 81, which is roughly 2.8 times the gain of the second stage. Finally, the fourth stage (d) yields the highest gain of approximately 90. This increasing trend in peak gain values across the stages suggests a progressive amplification of the signal, with each subsequent stage building upon the amplification provided by the previous stages to achieve a higher overall gain.

Supplementary Information 10

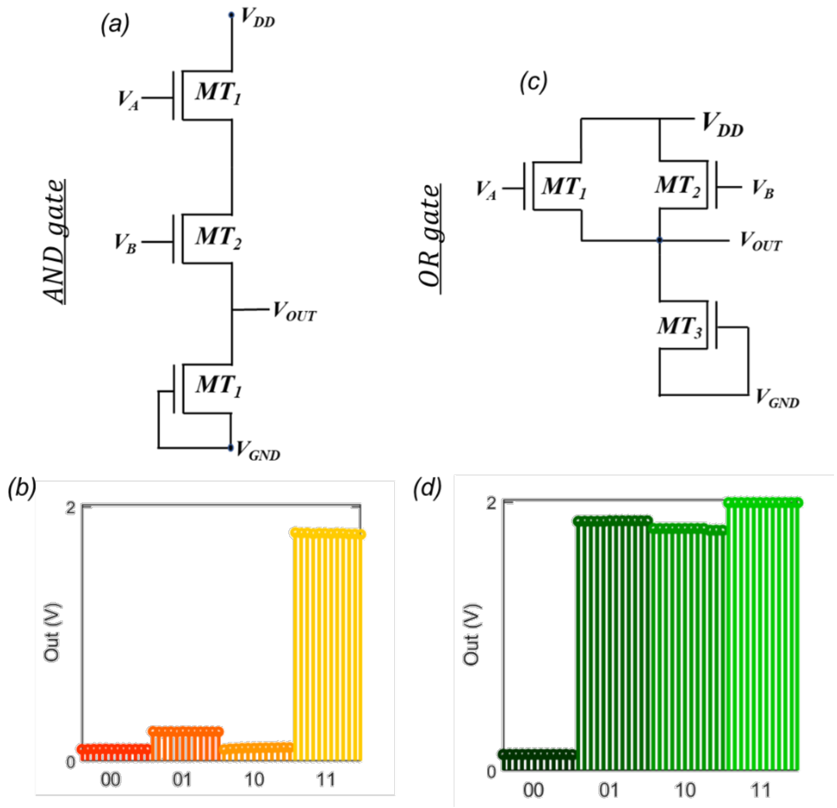

Supplementary Figure 10. Circuit schematic of an AND logic gate has been shown in (a). The AND gate is made of three representative 2D memtransistors, where the two input signals V_A and V_B are provided to MT_1 and MT_2, whereas MT_3 remains as a n-type depletion mode memtransistor. In this case, if either input, V_A or V_B, is at 0, the output of the AND gate, V_{Out}, will be consistently held at the lower logic level (~ 0 V) and V_{Out} only switches to the higher logic level or 2 V, when both the inputs are at 1 as shown in (b). Similarly, the circuit schematic of an OR logic gate is shown in (c), where MT_1 and MT_2 are connected in series with MT_3. Now, if the inputs to the memtransistors, V_A or V_B are both at 0, then only the output, V_{Out}, will be 0 V or logic low as shown in (d).

Supplementary Information 11

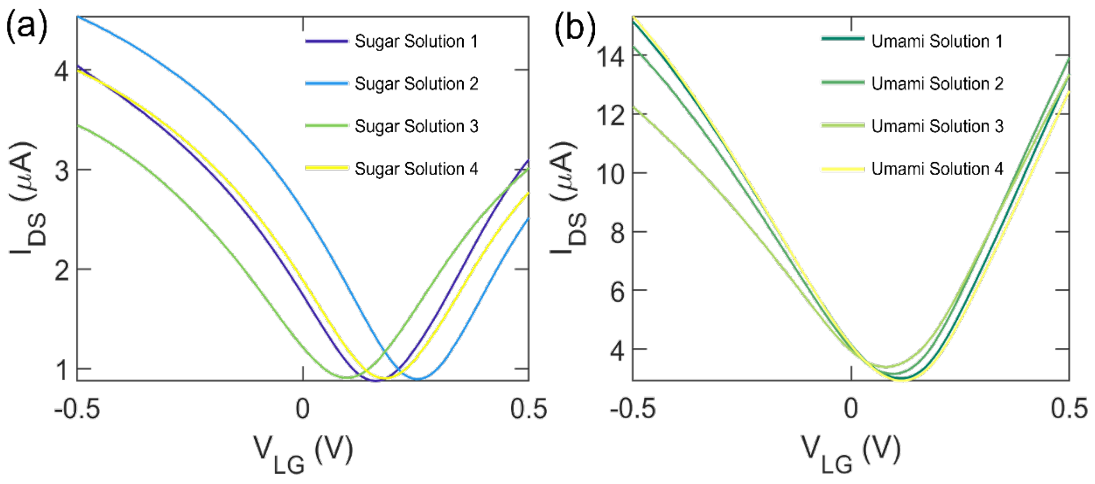

Supplementary Figure 11. Transfer characteristics of graphene chemitransistors for **a)** four different sugar solutions and **b)** four umami solutions at $V_{DS} = 10$ mV.

Supplementary Information 12

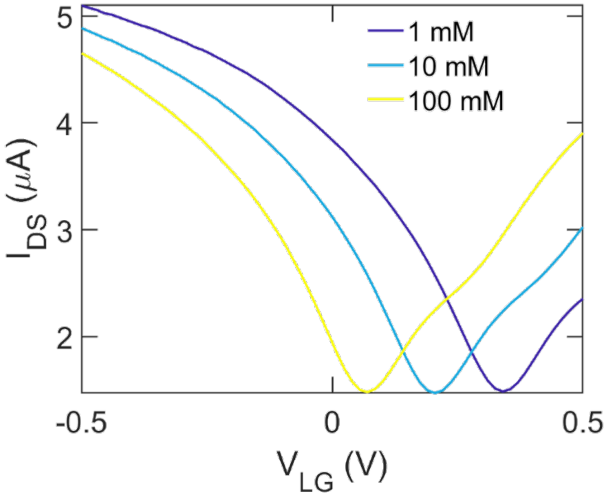

Supplementary Figure 12. Transfer characteristics of graphene-based taste receptors to different concentrations (1mM, 10mM, 100mM) of salt solution within a gate voltage range of -0.5V to 0.5V at $V_{\mathrm{DS}} = 10$ mV.

Supplementary Information 13

The concept of machines possessing emotional intelligence is an area of ongoing research and development [1, 2]. While machines cannot experience emotions in the same way humans do, incorporating emotional intelligence into their capabilities can offer several potential benefits especially with growing dominance of artificial intelligence (AI) in all spheres of our lives. Machines with emotional intelligence can understand and respond to human emotions, making interactions more natural and meaningful. Emotional intelligence also enables machines to understand individual needs and preferences. By recognizing emotions, they can provide tailored recommendations, suggestions, or support. For instance, a virtual assistant with emotional intelligence can detect if a person is stressed and recommend food that can uplift their mood [3]. Likewise, emotional intelligence can be applied in various ways within the food industry to enhance customer experiences and drive business success [4]. By incorporating emotional intelligence into the menu development process, restaurants can create dishes and experiences that evoke positive emotions, enhancing customer satisfaction and loyalty [5]. Emotional intelligence can also accelerate culinary innovation by understanding the emotional impact of different flavors, textures, and food combinations. While the benefits of machines having emotional intelligence are promising, we agree with the reviewer that it is essential to consider ethical considerations, data privacy, and the potential impact on human employment and relationships. Striking a balance between the benefits of emotional intelligence and responsible implementation is crucial for creating a positive and sustainable future with intelligent machines.

Supplementary Information 14

Our rationale for separating hunger and appetite is as follows: Hunger is a physiological sensation that arises from the body's need for nourishment. It is primarily driven by biological factors and the body's energy requirements. When you experience hunger, it is a signal from your body that it needs food to meet its energy needs and maintain proper function. Hunger is often accompanied by physical sensations like stomach growling, feeling lightheaded, or having low energy levels. Appetite, on the other hand, refers to the desire or preference for specific types of food or the urge to eat. Unlike hunger, which is primarily driven by biological factors, appetite is influenced by a combination of physiological, psychological, and social factors. Appetite is shaped by factors such as sensory cues (smell, taste, and appearance of food), learned preferences, emotional states, cultural influences, and environmental cues. Appetite can vary greatly from person to person and may not always align with actual physiological hunger. In summary, hunger is the physiological sensation of needing food to satisfy energy requirements, while appetite refers to the desire or preference for specific foods or the urge to eat.

However, we admit that it is not possible to completely separate hunger and appetite. In fact, the same is true for physiology and psychology and hence intellectual and emotional intelligence. As the physical connection between hunger and appetite becomes clearer from studies in psychology and biology, it will be possible in the future to refine the expression for feeding. However, considering the primary focus of this work, which is developing hardware components to capture different aspects of intelligence, we have decided to treat hunger and appetite as distinct concepts. Finally, we also acknowledge that hunger is primarily driven by body's internal energy requirements, whereas appetite can be driven by a combination of physiological, psychological, and social factors including sensory cues (smell, taste, and appearance of food), learned preferences, emotional states, cultural influences, and environmental cues. So, it is not fair to treat both as external stimuli. We also acknowledge that stimulating hunger in artificial systems can be challenging, as hunger is a complex physiological process. However, artificial systems can simulate hunger by monitoring and responding to specific physiological parameters associated with hunger. For example, tracking blood glucose levels, ghrelin (hunger hormone) levels, or even neural activity related to hunger signals can provide input for stimulating hunger in the system. However, considering the primary focus of the work, we have decided to treat hunger and appetite as external stimuli so that we can perform the proof-of-concepts experiments.

References

[1] B. A. Erol, A. Majumdar, P. Benavidez, P. Rad, K. K. R. Choo, and M. Jamshidi, "Toward Artificial Emotional Intelligence for Cooperative Social Human–Machine Interaction," *IEEE Transactions on Computational Social Systems,* vol. 7, pp. 234-246, 2020.

[2] D. Schuller and B. W. Schuller, "The age of artificial emotional intelligence," *Computer,* vol. 51, pp. 38-46, 2018.

[3] E. Brynjolfsson and A. Mcafee, "Artificial intelligence, for real," *Harvard business review,* vol. 1, pp. 1-31, 2017.

[4] I. Kumar, J. Rawat, N. Mohd, and S. Husain, "Opportunities of artificial intelligence and machine learning in the food industry," *Journal of Food Quality,* vol. 2021, pp. 1-10, 2021.

[5] K. Berezina, O. Ciftci, and C. Cobanoglu, "Robots, artificial intelligence, and service automation in restaurants," in *Robots, artificial intelligence, and service automation in travel, tourism and hospitality*, ed: Emerald Publishing Limited, 2019.
